# Emergency General Surgery and COVID-19 Pandemic: Are There Any Changes? A Scoping Review

**DOI:** 10.3390/medicina58091197

**Published:** 2022-09-01

**Authors:** Eleni Karlafti, Evangelia Kotzakioulafi, Dimitrios-Christos Peroglou, Styliani Gklaveri, Petra Malliou, Aristeidis Ioannidis, Stavros Panidis, Smaro Netta, Christos Savopoulos, Antonios Michalopoulos, Daniel Paramythiotis

**Affiliations:** 1 Emergency Department, University Hospital of Thessaloniki AHEPA, Aristotle University of Thessaloniki, 54636 Thessaloniki, Greece; 2 1st Propaedeutic Department of Internal Medicine, Medical School, AHEPA Hospital, Aristotle University of Thessaloniki, 54636 Thessaloniki, Greece; 3 1st Propaedeutic Surgical Department, University Hospital of Thessaloniki AHEPA, Aristotle University of Thessaloniki, 54636 Thessaloniki, Greece

**Keywords:** emergency general surgery, SARS-CoV-19 pandemic, comparison, COVID-19

## Abstract

*Background and Objectives*: The pandemic of SARS-CoV-19 has affected the overall spectrum of General Surgery, either in the case management part, or in the type of cases. The purpose of this review is to gather all the parameters affected and to compare these changes between the pandemic period and the corresponding time frame of the previous year. *Materials and Methods*: A review of literature in two electronic databases (PubMed and Scopus) was performed examining studies during the pre-pandemic (March to May 2019) and pandemic (March to May 2020) period about emergency surgeries. The differences in case presentation in emergency rooms, patient characteristics, length of hospitalization, type of surgery, complications and mortality rate were compared. *Results*: The comparison of the studies revealed significant results highlighting the differences between the two time periods for each parameter. There has been observed an overall decrease in the number of cases presented for emergency and urgent surgery. In terms of age, sex, and BMI, there were no significant variations amongst the patients. About the length of hospitalization, the patients hospitalized longer during the pandemic period. In terms of pathologies, the most common types of surgery were appendectomy, gastrointestinal, and colorectal resection. Mortality did not differ between the two study periods. *Conclusions*: COVID-19 affected a large part of Emergency General Surgery mainly concerns the type of operations performed. The hospitalization of patients, the complications that may have arisen and the recognition of emergencies were the most important issues faced by health care officials in hospitals during the period of COVID-19; however, there were parameters like mortality and patients’ characteristics that did not appear to differ with pre-pandemic era.

## 1. Introduction

The SARS-CoV-19 pandemic began in December 2019 in China, when a group of people with pneumonia of unknown cause was discovered and connected to a seafood market in Wuhan. A betacoronavirus, that was identified for the first time, called 2019 novel coronavirus, was discovered through unbiased sequencing and isolation of airway epithelial cells, and it became the seventh member of the coronavirus family infecting humans [1]. The World Health Organization (WHO) had proclaimed a Public Health Emergency of International Concern by the end of January 2020. The severe symptoms of COVID-19 have been connected to an increase in the number and rate of deaths, notably in China’s epidemic zone [2]. On 22 January 2020, the China National Health Commission released the details of the first 17 deaths [2].

Italy was the first European country to be seriously affected by the virus. Since the first case of SARS-CoV-19 was recorded on February 21st in the Lodi/Codogno area, the situation in Italy had been rapidly deteriorated, with the highest number of confirmed cases and deaths in the Northern areas, where the health system had been under tremendous stress. Following China’s lead, Italy, as most of the European countries, took a variety of social distancing measures, varying from social distancing at first to a complete lockdown extended to the whole country [3].

As a result of the COVID-19 pandemic our lives have been drastically changed and, particularly, the pandemic had a profound long-term impact on healthcare services all over the world. To adjust to the rising number of emergency admissions for respiratory syndromes, the majority of which required intensive care, every healthcare system experienced considerable changes. To deal with this unprecedented disaster, each country devised its own guidelines and procedures.

Even though most elective services had been stopped, the emergency cases had to be treated anyway. In particular, general surgery’s acute abdomen admissions and trauma continued to need emergency treatment as a necessity. Acute appendicitis and gallbladder disorders were two of the most common reasons for seeking urgent care and emergency medical treatment [4].

With this review, we aim to examine the influence of the SARS-CoV-19 pandemic on the surgical domain, especially in Emergency General Surgery. We summarized the results of 23 studies and articles concerning the differences in presented cases in emergency operation rooms, the characteristics and comorbidities of the patients included in the studies, the length of hospitalization, the type of surgery, the complications and the mortality rate.

## 2. Methods

This review examines the changes in Emergency General Surgeries during the pandemic in comparison with the pre-COVID era. According to Centers for Disease Control and Prevention (CDC), 42 states and territories were under mandatory stay-at-home commands from 1 March to 31 May (2020) during the start of the pandemic of SARS-CoV-19, having an impact on 2355 (73%) of 3233 U.S. countries [2,3,4,5,6,7,8]. Mainly, the months under investigation, were March to May 2019 and 2020. We defined the time frame of March-May 2019 as the ‘pre-pandemic period’ or ‘pre-pandemic era’, which is the respective era of the previous year to the SARS-CoV-19 pandemic period and the time of March–May 2020 as the ‘pandemic group’ and ‘pandemic era’ throughout the whole following text.

A thorough literature search in 2 electronic databases, PubMed and Scopus was conducted in March 2022 with the following search terms: ‘surger*’ AND ‘emergency’ AND ‘emergency surger*’ AND ‘pandemic’ between 1 January 2020 and 9 March 2022.

For an article to be eligible should be published in this time frame, written in English, mentioning emergency surgeries and provide data regarding presented cases in emergency operation rooms, the characteristics and comorbidities of the patients included in the studies, the length of hospitalization, the type of surgery, the complications and the mortality rate.

The selected articles regard the comparison between the time frame of the COVID-19 ‘wave’ and the respective era of the previous year. The prime objective of this review is to find out if emergency case procedures in General Surgical departments have changed during the mentioned period; this review is focused mainly on the general surgery specialty and its subspecialties.

**Figure 1:**
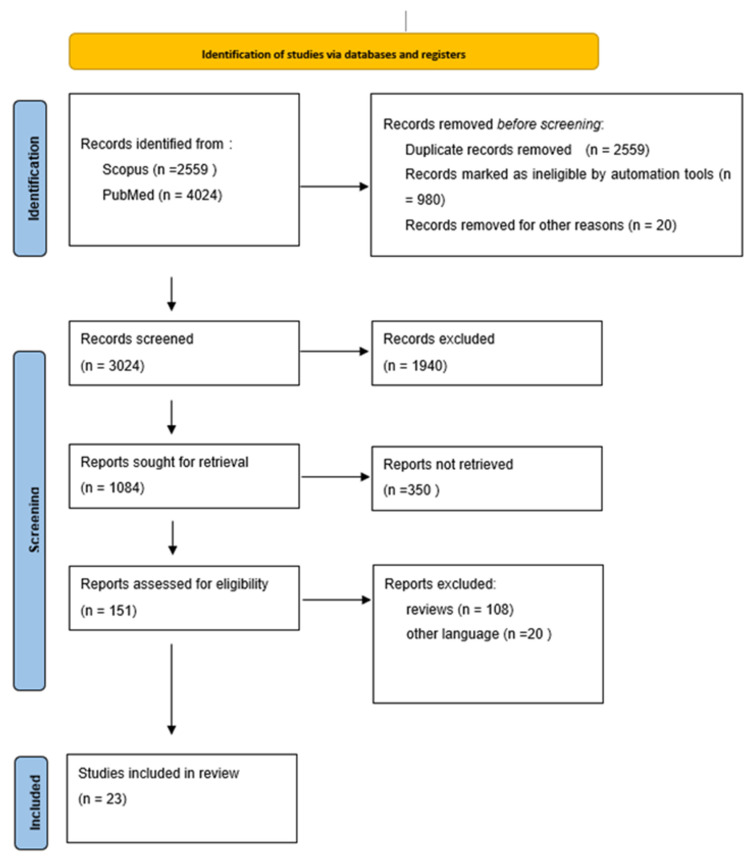
PRISMA Flow chart.

All resulting studies were screened by D.C.P., S.G., P.M., A.I., S.P. and S.N., and eligible articles were recorded using standardized data forms in Microsoft Excel in order to present the data more clearly. Results of the literature search is presented in Figure 1. All available data are presented in Table A1, Table A2 and Table A3 in Appendix A [5,6,7,8,9,10,11,12,13,14,15,16,17,18,19,20,21,22,23,24,25,26,27,28].

## 3. Results

### 3.1. Characteristics of Studies

We summarized the results of 23 studies and articles concerning the differences in presented cases in emergency operation rooms, the characteristics and comorbidities of the patients included in the studies, the length of hospitalization, the type of surgery, the complications and the mortality rate. As is shown in Table A2 there were no significant differences in age and gender of patients presented in emergency departments.

### 3.2. Differences in Presented Cases in Emergency Operation Rooms

In the scoping of literature, there has been observed an overall decrease in the number of the cases presented for emergency and urgent surgery in the field of General Surgery between the two periods. According to S. Wades et al. the biggest single drop in emergency caseload was recorded in General Surgery which, along with trauma and orthopedics, accounted for the majority of patients handled at their trust; this discovery was not singular; in the month after the Italian government’s lockdown order, a study involving three large hospitals in northern Italy reported an 86% decrease in surgical emergencies [6]; as also seen in Germany [9] this phenomenon of a decrease in the activity of surgeries on a global scale had not previously been observed in studies other than two specific papers referring to the period of the Ebola epidemic [7].

However, there have been some studies with inconsistent results regarding the number of surgeries during the pandemic. For instance, H. Drysdale et al. observed an increase of 13.9% in emergency laparoscopic cholecystectomies and emergency colonoscopies that had been performed. There were no significant changes in laparoscopic cholecystectomies [10].Two more studies on surgical emergencies in Greece and Italy observed decrease in emergency surgeries [11,12].

According to G. van Aert’s et al. research, the rise in acute emergency trauma surgery on the elderly is linked to prescribed measures for isolation, resulting in reduced family and nurse attendance for the elderly, increasing the danger of falling [13]. The number of patients who underwent emergency abdominal surgery at Aizawa Hospital (Matsumoto, Japan) was indifferent between the two study periods [17]. Furthermore, emergency reconstruction surgery for hernias showed an increase [27].

The results from four studies showed that the average duration of symptoms prior to presenting to the ERs (Table A3) was increased, either due to difficult assessments in acute medical services as a result of the strict lockdown measures or bigger consideration of COVID-19 risk patients who presented significantly later after the onset of their symptoms [7,9,24,25]. In contrast, the study of Y. Nishida et al. concluded that this time remained unchanged [17].

For the time that elapsed between hospital admission and surgery (Table A3) was greater in the pandemic era compared to the pre-pandemic period [5]. Even though the mean delay of the surgical procedure was increased, the percentage of people who were operated on the day of their admission (urgent surgery, 24 h) did not vary significantly. [13].

### 3.3. Patient’s Characteristics—Comorbidities

We summarized the basic patient characteristics involved in the majority of the studies.

We scoped via data searching (Table A1, Table A2 and Table A3). In terms of age, gender, and BMI, we compared the demographic data from the two groups. For age, we estimated the mean age of both study groups and found that during the pre-pandemic era (2019) and the pandemic period (2020), these parameters for patients undergoing emergency procedures were 50.9 years and 49.5 years, respectively, noting no significant difference.

Regarding sex, most emergency surgeries were slightly more prevalent in males, with the exception of two particular types of surgical emergency pathological disorders, acute appendicitis and diverticulitis, which were more common for female patients [20,21]. Regarding BMI, the majority of patients were normal or overweight, with no quantitatively significant difference between the two periods [8,22,23].

In the studies, there was a small percentage of patients who had comorbidities. The most prevalent comorbidity was hypertension [7,8,22]. Other comorbidities most commonly included were dyslipidemia, diabetes mellitus, cardiovascular diseases and COPD. According to Zoilo Madrazo et al. study’s patients who were positive for SARS-CoV-19, they had an extra 10% of people with comorbidities in the pandemic period (2020), whereas SARS-CoV-19 negative patients and patients from 2019 showed no difference in the percentages of patients who had comorbidities [22].

### 3.4. Length of Hospitalization

Patients were hospitalized longer during the pandemic period than those during the pre-pandemic era [7,8,16] (Table A3). There was no change in hospital stay duration between the two periods for patients who did not contract COVID-19 [8]. The mean days of hospitalization among the different types of surgeries during the pre-COVID-19 and pandemic era showed an increase in Abdominal surgeries, Hernia surgeries and a decrease in Hepatobiliary surgeries [11]. The results of the two studies showed no differences in length of stay [9,16].

### 3.5. Type of Surgeries According to Pathological Status and Surgical Approach

During the pre-pandemic era, the most prevalent types of surgery regarding the pathologies were appendicectomy, gastrointestinal and colorectal resection due to obstruction, hernia reparation and soft tissue infection [5,7,11,24]. Casella et al. reported that the most common types of surgery in their hospital during the pandemic period were GI and colorectal resection (25%), abscess drainage (18.8%), and adhesiolysis (18.8%).

Acute appendicitis revealed no significant difference in case presentation, despite a decrease in the number of procedures [7,15,24,25,26]; it is worth noting that the pandemic group had a significantly greater rate of complicated appendicitis [7,15]. Karlafti et al. noted that the most frequent types of surgery were hepatobiliary surgeries and soft tissue surgeries in the pandemic era [11].

For bowel obstruction, there has been a significant increase in the number of surgeries but Surek et al. observed a reduction [7,12,15,24]. As regards acute cholecystitis, there has been an overall decline in the number of surgeries that have been performed [7,15,24]. Although, an increase was noticed on the laparoscopic approach for cholecystectomies during the pandemic era [10,19]. Surek et al. found a 92% reduction in surgeries regarding hernias reparation [15]. On the other hand, Cano-Valderramaa et al. observed a minor increase in the percentage of hernia surgeries [7].

### 3.6. Complications

The majority of studies showed no statistically significant difference in complications between the two mentioned periods (Table A3) [8,9,15,16,21,25]. D’Urbano et al. found a substantial increase in complications for the pandemic group but noted that the number of patients involved in the study was small and could not be statistically relevant [19]. Casella’s et al. results, which described an increase, were likewise inconsistent. Kamil et al. observed that for patients with acute appendicitis who underwent appendectomy, there was a statistically significant rise compared with the Clavien-Dindo morbidity scale between the two groups [25].

### 3.7. Mortality

There was no difference in fatality rates between the pre-pandemic and pandemic group in most studies [7,8,10,11,18,23]; however, certain studies show considerable discrepancies when compared to the plurality of other studies (Table A3). For instance, G. Casella et al. discovered a statistically significant increase in mortality [5]. In the study by A. Surek et al., the pandemic group had a marginally higher fatality rate. [15]. Z. Madrazo et al. reported 30-day mortality as statistically greater in a number of patients, yet there were individuals positive for SARS-CoV-19 were included in the pandemic group [22]. F. D’Urbano et al. reported a minor decrease in mortality rates; however, as we previously stated, their findings are not statistically significant because of the small number of patients in their research [19].

## 4. Discussion

The COVID-19 pandemic posed an unprecedented challenge for medical professionals all across the world, especially during the first wave of SARS-CoV-19 during the pandemic period, when knowledge about the new strain of the virus was little. Emergency General Surgery was one of the surgical specialties that was severely impacted, considering the fact that it is a sector in which a vast amount of emergency cases are being treated in everyday medical practice.

In Greece, emergency general surgeries took a stand and dealt with a burdenous situation. Due to a lack of hospital beds, many operating rooms reverted to hospital beds with enhanced care for patients, therefore, resulting to emergencies ran late for appropriate care. As the pandemic withholds, many patients are not provided with appropriate care or even delay their diagnoses; these circumstances may appeal to other countries also but as smaller a healthcare system is, the bigger these disparities will arise.

In the field of Emergency General Surgery internationally, we found that there were disparities but not majorly differences in the number of patients who arrived for emergency or urgent surgery, the length of symptoms, the period between admission and operation, and the types of surgeries performed. We also must mention that there is lack of data in order to provide more clear results. Nonetheless, we observe an increase in duration of symptoms before asking for care, inconsistent data regarding length of stay and no change in the time between admission and surgery. There were no considerable differences in patient’s characteristics, complications or fatality between the two periods that this review examines; this means that the pandemic influenced mostly the perception of patients and delayed them from seeking care in freight of COVID-19. Nevertheless, their delay hopefully did not result in more deaths or complications.

As is shown in the past, Koutserimpas et al., in their analysis showed that emergencies in economic crisis rise but admissions decreased [29]; this also was shown in pediatric patients by Gkentzi et al., where children came more to the hospital with fever or respiratory disorders but this did not affect admissions rate [30]. Regarding diagnosis, periods of economic instability is a predictor for a delayed diagnosis and there is a need for more access to primary healthcare [31]. What is more, Karavokyros et al., presented an increase in emergency inguinal hernioplasty and more prevalent in younger patients in an economic crisis time in Greece, addressing the redistribution of surgical workload in urban hospitals [32]; this is in concordance with our findings that more younger patients needed emergency surgeries but came late in the emergency department.

The limitations of this review are the scarce and little available data regarding emergency general surgeries during the COVID-19 pandemic and the lack of known registries regarding surgeries throughout the world. One more limitation of our study is that we did not proceeded with meta-analysis. Possibly, a meta-analysis could provide a more clear result of the impact of the pandemic in the field. Therefore, we proceeded to a scoping review because the data were not sufficient enough to meta-analyse. The strengths of our review are that it is the first review about the change in emergency general surgery during COVID-19.

All these findings suggest improving access to healthcare systems for all patients, especially during hard times and the need for more hospitals and primary health centers that will aid in better and earlier diagnoses.

## 5. Conclusions

COVID-19 was an unprecedented challenge for medical professionals and healthcare systems throughout the world which severely impacted also Emergency General Surgery. Our findings suggest a slight difference in age of patients seeking care and delay but all these did not conclude to more complications or increased mortality; this crisis should act as a start for the better adaptation of healthcare systems during crisis.

## Data Availability

Not applicable.

## Appendix A

**Table A1:** Summary of findings for the included studies.

Study ID	Diagnoses		Results
	Pre	Pan	
Balla et al.	Bowel ischemia (0%), Bowel occlusion (12%), Bowel perforation (12%), Abdominal trauma (4%), Appendectomy (16%), Cholecystectomy (12%), Abdominal wall (4%), Pneumothorax (16%), Urology (12%), Other (12%)	Bowel ischemia (11.8%), Bowel occlusion (11.8%), Bowel perforation (17.6%), Abdominal trauma (5.8%), Appendectomy (11.8%), Cholecystectomy (11.8%), Abdominal wall (11.8%), Pneumothorax (5.8%), Urology (11.8%), Other (0%)	There were no differences in emergency general surgeries.
Cano-Valderrama et al.	Acute appendicitis (30.9%), Perianal abscess (12.6%), Acute cholocystitis (12.3%), Complication of an elective procedure (14.4%), Complication of an elective procedure (14.4%), Bowel obstruction (6.7%), Abdominal wall hernia (5.6%), Other (17.5%)	Acute appendicitis (34.2%), Perianal abscess (14.5%), Acute cholocystitis (2.6%), Complication of an elective prosedure (6.8%), Bowel obstruction (12.8%), Abdominal wall hernia (9.4%), Other (19.7%)	There was a change in the gender (M > F) during pandemic period. Avarage duration of symptoms prior to presenting in the emergency department was increased (*p* < 0.001).
Carpio Colmenares et al.	Acute appendicitis (55.5%), Acute lithiasic cholecystitis (37.7%), Hernias (6.7%), Gastric perforation (0%), Intestinal obstruction (0%)	Acute appendicitis (50.8%), Acute lithiasic cholecystitis (42.4%), Hernias (3.4%), Gastric perforation (1.7%), Intestinal obstruction (1.7%)	In this study there was an increase in length of stay. The degree of severity of emergency abdominal surgical pathology in pandemic has not increased, except for post operative complications.
Casella et al.	Appendicectomy (17%), Gastrointenstinal or Colorectal resections (18.2%)	Gastrointenstinal or Colorectal resections (25%), Abscess drainage (18.8%), Adhesiolesis (18.8%)	The time elapsed between hospital admission and surgery was increased. The percentage of patients who experience post operative complications was increased, *p* = 0.019. The mortality was increased (*p* < 0.0001)
D’Urbano et al.	Cholecystectomy (21.7%), Appendectomy (19.7%), Hemothorax/Pneumothorax/Pleural effusion (8.6%), GI perforation (10.9%), Bowel obstruction (15.2%), Bowel infraction (6.5%), Hemoperitoneum evacuation (6.5%), GI bleeding (2.2%), Fasciotomy/Abcess drainage/Hematoma drainage (6.5%), Other (2.2%)	Cholecystectomy (22.2%), Appendectomy (0%), Hemothorax/Pneumothorax/Pleural effusion (14.9%), GI perforation (11.1%), Bowel obstruction (33.3%), Bowel infraction (3.7%), Hemoperitoneum evacuation (0%), GI bleeding (3.7%), Fasciotomy/Abcess drainage/Hematoma drainage (11.1%), Other (0%)	D’Urbano et al. found a reduction in the number of patients who were hospitalized and underwent emergency surgery. Mean age was higher in the pre-pandemic period. The complications were increased in the pandemic era in 2020. The fatality rate after surgery decreased.
Drysdale et al.	Laparoscopic appendicectomy (50%), Soft tissue infection (41%), Laparoscopic cholecystectomy (26%), Laparotomy (22%), Hernia repair (8%), Gastroscopy (16%), Colonoscopy (7%), ERCP (7%)	Laparoscopic appendicectomy (48%), Soft tissue infection (46%), Laparoscopic cholecystectomy (40%), Laparotomy (21%), Hernia repair (7%), Gastroscopy (5%), Colonoscopy (13%), ERCP (6%)	Drysdale et al. reported a 13.9% rise in emergency laparoscopic cholecystectomies and emergency colonoscopies; this study did not found any other differences between the two periods.
Fallani et al.	Secondary peritonitis	Secondary peritonitis	Patients who underwent surgery during pandemic had a higher rate of severe peritonitis. The surgery lasted longer in the pandemic period. The mean time elapsed between hospital admission and surgery was greater in the pandemic period. In the pandemic era there was a bigger proportion of secondary peritonitis caused by appendicitis and a smaller proportion caused by perforated peptic ulcer. Complications and length of stay were increased in the pandemic group.
Fowler et al.	-	-	A decline was seen in the proportion of emergency procedures in general surgery; this study says the total number of procedures did not significantly decreased in emergency general. surgery.
Hossain et al.	Acute diverculitis	Acute diverculitis	There was a decrease in the cases of Acute diverculitis. The proportion of patients who underwent emergency surgery was significantly higher during the pandemic period (*p* = 0.04).
Kamil et al.	Cholecystitis, Appendicitis, Diverculitis	Cholecystitis, Appendicitis, Diverculitis	Hospital admissions decreased in March and increased in April and May. The treatment method was primarily conservative in 2020.
Karlafti et al.	Digestive system surgeries (19.7%), Hernia repair (22.6%), Soft tissue infection surgeries (28.3%), Hepatobiliary surgeries (21.7%)	Digestive system surgeries (16.6%), Hernia repair (18.4%), Soft tissue infection surgeries (26%), Hepatobiliary surgeries (30.5%)	During the pandemic period, mortality rates nearly doubled (2.2% vs. 4%), although the total number was significantly lower than during the control period.
Kumaira Fonseca et al.	Acute appendicitis	Acute appendicitis	The cases were lower in the pandemic period. The average time of symptom onset to arrival at the emergency department was higher in the pandemic. There was a higher proportion of complicated cases during the pandemic.
Madzaro et al.	Complexity: Minor (749)/Moderate (1311)/Major (848)/Major+ (82)	Complexity: Minor (508)/Moderate (1081)/Major (712)/Major+ (57)	There were differences in complications in the lenght of stay and in post-operative mortality; this study finds post-operative mortality of COVID-19 positive patients was greater for Minor, Moderate, Major and Major+ procedures.
Malik et al.	Hernia repair	Hernia repair	There was an 18% increase in the number of surgeries for Hernia reparation in the pandemic era. The duration of symptoms prior to presenting in the ER was slightly increased in 2020. In this study is mentioned that the pandemic resulted to cancellations of non-urgent elective procedures for hernias and that is why emergency hernia operations were increased in the pandemic era, considering the acute presentation of symptomatic hernias.
Osorio et al.	-	-	This multi-centered study concluded no differences in the results between the two periods, but points out that patients infected with SARS-CoV-19 had worse outcomes after the surgery.
Rashdan et al.	Acute appendicitis, Acute cholecystitis, Acute pancreatitis, Intestinal obtruction, Complicated hernia, For observation, Perianal pain, Soft tissue infection, Burn, Trauma, Others	Acute appendicitis, Acute cholecystitis, Acute pancreatitis, Intestinal obtruction, Complicated hernia, For observation, Perianal pain, Soft tissue infection, Burn, Trauma, Others	Admissions decreased. There were more male patients in the pandemic group. Duration of symptoms before the ER visit was longer in the pandemic.
Rausei et al.	Appendicitis, Cholecystitis, Bowel obstruction, Bowel perforation, GI bleeding, Proctologic diseases, Abdominal trauma	Appendicitis, Cholecystitis, Bowel obstruction, Bowel perforation, GI bleeding, Proctologic diseases, Abdominal trauma	Emergency surgical admissions and surgical operations significantly decreased from March 2019 to March 2020, no other changes found.
Salgaonkar et al.	Acute appendicitis	Acute appendicitis	The only differences on appendicectomy was that percentage of surgical site infections was increased in the pandemic period.
Surek et al.	Trauma (11), GI bleeding (9), Acute mesenteric ischemia (2), Perforations (14), Acute mechanical intestinal obstruction (25), Incarcerated hernia (25), Acute cholecystitis (55), Acute appendicitis (155)	Trauma (12), GI bleeding (7), Acute mesenteric ischemia (2), Perforations (18), Acute mechanical intestinal obstruction (24), Incarcerated hernia (2), Acute cholecystitis (29), Acute appendicitis (42)	There was a 59.1% reduction in the number of emergency surgeries and 50% decrease in the number of non-operatively followed patients in the pandemic group. There was a 47.3% and a 73% reduction in the number of patients who had surgery for acute cholecystitis and for acute appendicitis, respectivly.
van Aert et al.	Minor trauma (2.5%), Major trauma (17.9%), Polytrauma (5.6%), Neck of Femur (53.7%), Soft tissue trauma (3.7%), Pediatric trauma (16.7%)	Minor trauma (8.1%), Major trauma (21.4%), Polytrauma (4.6%), Neck of Femur (50.3%), Soft tissue trauma (5.8%), Pediatric trauma (9.8%)	There was an overall decrease in trauma-related admissions. The age was significantly higher in 2020 with fewer adolecents and more senior patients. In 2020, more patients underwent minor surgery. Comparingly in 2020 there was a bigger proportion of patients falling from standing high than 2019; furthermore, trauma-related surgeries were increased in 2020. There was a reduction of the number of car and motorcycle accidents.
Wade et al.	-	-	-
Wilms et al.	Appendicitis	Appendicitis	The overall number of patients decreased. Avarage duration of symptoms prior to presenting in the emergency department was increased.
Yasunori Nishida et al.	Acute appendicitis (45%), Acute cholecystitis (12%), Strangulated small bowel obstruction (5%), Colon perforation (10%), GI perforation (11%), Malignant bowel obstruction (4%), Others (8%)	Acute appendicitis (41%), Acute cholecystitis (18%), Strangulated small bowel obstruction (14%), Colon perforation (1%), GI perforation (5%), Malignant bowel obstruction (6%), Others (4%)	This study found not statistically significant differences of the involved parameters of our study.

Abbreviations: pre: Pre pandemic period, pan: pandemic period.

**Table A2:** Summary findings with characteristics of patients of the included studies.

	Age (Years)			Gender			BMI		
Study ID	Pre	Pan	*p*	Pre	Pan	*p*	Pre	Pan	*p*
Cano-Valderrama et al.	55	52.6	0.276	F (50.88%)	F (33.8%)	0.001			
D’Urbano et al.	65	63.5		M (54.3%)	M (55.5%)				
Fallani et al.	44	49	0.223	M (53%)	M (63.1%)	0.065	25.2 ± 2.4	25.3 ± 2.1	0.675
Hossain et al.	63.3	62.6	0.762	M (47.7%)	M (46.2%)	0.867			
Karlafti et al.	51.2 ± 17.6	49.3 ± 17.1		M (53.9%)	M (58.2%)	0.284			
Kumaira Fonseca et al.	34.3 ± 5.8	38.2 ± 18.1	ns	F (62.2%)	F (55.6%)	Ns			
Osorio et al.	57	56		M (58.6%)	M (59.8%)		27.3	27.3	ns
Surek et al.	46.1 ± 17.9	46.6 ± 18.9	0.890	M (63.5%)	M (65%)	0.928			
Wilms et al.	35 ± 19	36 ± 20	0.24	M (N:510)	M (N:468)	0.18			
				F (N:517)	F (N:420)				
van Aert et al.	42	48	>0.001	F (47.8%)	F (52%)	0.088			

Abbreviations: M: male, F: female, ns: non-significant, pre: Pre pandemic period, pan: pandemic period.

**Table A3:** Results of the included studies.

Study ID	Country	Publication Year	Study Period	Patients Included		Number of Surgeries		Average Duration of Symptoms Prior to Presenting in Emergency		Time (Hours) Elapsed between Hospital Admission and Surgery		Length of Stay (Days)		Complications % of pts Experiencing Complications		Mortality	
				Pre	Pan	Pre	Pan	Pre	Pan	Pre	Pan	Pre	Pan	Pre	Pan	Pre	Pan
Balla et al.	Italy	Junuary 2021	8/3–4/5/2019–8/3–4/5/2020	99	41	25	17										
Cano-Valderrama et al.	Spain	July 2020	11/3–21/4/2019–11/3–21/4/2020	285	117	285	117	**44.6 h**	**71 h**	12.4 (N: 285)	12.3 (N: 117)	12.2	8.5			4.27%	6.67%
Carpio Colmenares et al.	Peru	2021	11/3–8/6/2019–11/3–8/6/2020	45	59	45	59					**1.73 ± 1.07**	**2.74 ± 2.80**				
Casella et al.	Italy	February 2022	9/3–9/5/2019–9/3–9/5/2020	476	79	88	16			**16.73 ± 1.76 (N: 88)**	**22.56 ± 11.12 (N:16)**					**0%**	**31.3%**
D’Urbano et al.	Italy	September 2020	9/3–9/4/2019–9/3–9/4/2020	46	27	46	27							36.9%	55.5%	19.6%	11.1%
Drysdale et al.	Australia	July 2020	1/4–19/5/2019–30/3–17/5/2020	506	475	180	205									1 pt	1 pt
Fallani et al.	Italy	December 2020	23/3–4/5/2019–23/3–4/5/2020	183	149	183	149							**18%**	**35.6%**	4.9%	6%
Fowler et al.	Australia	December 2020	1/3–24/4/2019–1/3–24/4/2020	1574	1240	694	596										
Hossain et al.	UK	2020	1/3–30/6/2019–1/3–30/6/2020	107	52	1	4										
Kamil et al.	Ireland	June 2021	1/3–31/5/2019–1/3–31/5/2020	138	94	84	33	2.70 ± 2.86 days	3.13 ± 2.67 days								
Karlafti et al.	Greece	November 2021	3/2019–2/2020–3/2020–2/2021	456	223	456	223					4 ± 8.6	4.6 ± 10.3			2.2%	4%
Kumaira Fonseca et al.	Brazil	2020	March–April 2019–March–April 2020	82	36	82	36							13.4%	11.1%		
Madzaro et al.	Spain	November 2021	1/3–30/6/2019 1/3-30/6/2020	2800	2188	2800	2188									<0.001	
Malik et al.	UK	June 2021	1/1–31/12/2019–1/1–31/12/2020	32	39	32	39										
Osorio et al.	Spain	September 2021	1/3–30/6/2019–1/3–30/6/2020	2992	2315	2992	2315					4	4			3.2%	5.2%
Rashdan et al.	Jordan	May 2021	March–June 2019–March–June 2020	201	143	154	60	**57 ± 64.4 min**	**95.32 ± 148.62 min**								
Rausei et al.	USA	August 2020	3/2019–3/2020	869	475	515	302										
Salgaonkar et al.	UK	March 2021	1/3–5/6/2019–1/3–5/6/2020	206	132	206	132									0%	0.7%
Surek et al.	Germany	November 2020	14/3–15/5/2019–14/3–15/5/2020	453 (total in both)	453	252	103							17.06%	24.27%	**1.19%**	**4.85%**
van Aert et al.	The Netherlands	February 2021	11/3–10/5/2019–11/3–10/5/2020	1717	1182	162	173			(N:162)	(N:173)						
Wade et al.	England	June 2020	23/3–10/5/2019–23/3–10/5/2020	193	64	-	-										
Wilms et al.	Germany	Janruary 2021	February–March/2019–February–March/2020	1027	888	1027	888	37.5 ± 45.8 h	41.1 ± 55.9 h			4.5 ± 4.1	4.6 ± 4.0				
Yasunori Nishida et al.	Japan	December 2020	1/3–30/6/2019–1/3–30/6/2020	90	89	90	89										

Results in bold were statistically significant (*p* < 0.05).
